# Using ultrasound features and radiomics analysis to predict lymph node metastasis in patients with thyroid cancer

**DOI:** 10.1186/s12893-020-00974-7

**Published:** 2020-12-04

**Authors:** Fu Li, Denghua Pan, Yun He, Yuquan Wu, Jinbo Peng, Jiehua Li, Ye Wang, Hong Yang, Junqiang Chen

**Affiliations:** 1 Department of Gastrointestinal Surgery, First Affiliated Hospital of Guangxi Medical University, 6 Shuangyong Road, Nanning, 530021 Guangxi Zhuang Autonomous Region People’s Republic of China; 2 Department of Ultrasonography, First Affiliated Hospital of Guangxi Medical University, 6 Shuangyong Road, Nanning, 530021 Guangxi Zhuang Autonomous Region People’s Republic of China

**Keywords:** Thyroid carcinoma, Lymph node metastasis, Ultrasound, Radiomic analysis

## Abstract

**Background:**

Lymph node metastasis (LNM) is an important factor for thyroid cancer patients’ treatment and prognosis. The aim of this study was to explore the clinical value of ultrasound features and radiomics analysis in predicting LNM in thyroid cancer patients before surgery.

**Methods:**

The characteristics of ultrasound images of 150 thyroid nodules were retrospectively analysed. All nodules were confirmed as thyroid cancer. Among the assessed patients, only one hundred and twenty-six patients underwent lymph node dissection. All patients underwent an ultrasound examination before surgery. In the radiomic analysis, the area of interest was identified from selected ultrasound images by using ITK-SNAP software. The radiomic features were extracted by using Ultrosomics software. Then, the data were classified into a training set and a validation set. Hypothetical tests and bagging were used to build the model. The diagnostic performance of different ultrasound features was assessed, a radiomic analysis was conducted, and a receiver operating characteristic (ROC) curve analysis was performed to explore the diagnostic accuracy.

**Results:**

Regarding the prediction of LNM, the ROC curves showed that the area under the curve (AUC) values of an irregular shape and microcalcification were 0.591 (P = 0.059) and 0.629 (P = 0.007), respectively. In the radiomics analysis, in the training set, the AUC value of LNM was 0.759, with a sensitivity of 0.90 and a specificity of 0.860. In the verification set, the AUC was 0.803, with a sensitivity of 0.727 and a specificity of 0.800.

**Conclusions:**

Microcalcification and an irregular shape are predictors of LNM in thyroid carcinoma patients. In addition, radiomics analysis has promising value in screening meaningful ultrasound features in thyroid cancer patients with LNM. Therefore, the prediction of LNM based on ultrasound features and radiomic features is useful for making appropriate decisions regarding surgery and interventions before thyroid carcinoma surgery.

## Background

Thyroid nodules are frequently found by clinicians, but thyroidectomy is needed for only a subset of thyroid nodules. Most types of thyroid cancer are described as indolent carcinomas that have a good prognosis [1]. However, recurrence and metastasis are still unavoidable, especially in undifferentiated thyroid cancer. Cervical lymph node metastasis (LNM), which occurs in regions of the neck, is a common type of metastasis of thyroid cancer, with a frequency of 30% to 90% in papillary thyroid cancer [2]. In addition, LNM is an important factor for recurrence [3–6]. The revised American Thyroid Association management guidelines indicate that LNM in the central region of the neck is considered a risk factor in thyroid cancer [7]. In addition, LNM has an important reference value in the determination of surgical plans.

Ultrasonography, including greyscale ultrasonography, colour Doppler ultrasonography, ultrasound elastography, contrast-enhanced ultrasound and ultrasound-guided fine-needle aspiration biopsy (FNAB), has been widely applied to screen thyroid nodules and LNM. Studies have reported that suspicious ultrasound features are associated with LNM [8, 9]. However, the sensitivity of LNM detection by ultrasound is low at approximately 41.3%-61% [10–12]. FNAB is an invasive method used to identify nodules and is unnecessary in most patients [13]. Therefore, identifying an effective and noninvasive way to detect lymph nodes by radiologists is necessary.

Radiomics analysis was first reported by Lambin P in 2012 [14]. This analysis is mainly applied to extract and analyse imaging features from medical images, such as computed tomography (CT) and magnetic resonance images, to quantitatively evaluate diseases [15]. Studies have shown that image feature-based radiomics extraction has objective characteristics and great value in predicting clinical outcomes [14]. Radiomics analysis has been applied in various diseases, such as thyroid cancer [16], lung cancer [17], liver cancer [18] and breast cancer [19]. Therefore, the use of ultrasound radiomics analysis to predict lymph node metastasis before surgery may have an important clinical value.

The purpose of our study was to explore the correlations between ultrasound features and LNM and further develop a radiomics analysis method for the prediction of LNM in thyroid cancer before surgery.

## Methods

### Patients

This retrospective study was approved by the Ethics Committee of the First Affiliated Hospital of Guangxi Medical University. One hundred and fifty patients were confirmed to have thyroid cancer, and lymph node sections were obtained in our hospital from August 2016 to December 2018. Only one hundred and twenty-six patients were included in the radiomic analysis. These 126 patients included 94 females and 32 males. The mean age was 38.06 years (range, 17–82 years). All patients signed an informed consent form discussing the use of the patients’ information for scientific research to the full extent of the law before they were admitted to the hospital. The inclusion criteria were as follows: patients who underwent surgical resection within 2 weeks after a neck ultrasound examination; patients with pathologically confirmed thyroid cancer; patients who underwent lymph node resection; and patients with a confirmed lymph node state. The exclusion criteria were as follows: patients who underwent preoperative radiotherapy or radiation therapy and patients who had unclear ultrasound images.

### Greyscale ultrasonography examination

All included patients underwent an ultrasound examination before surgery. The ultrasound examination was performed using a GE LOGIQ E9 ultrasound system with a 6-15L linear array probe, and the frequency was set to 11–13 MHz. An Esaote system (MYLAB CLASS C) with an 11-L4 linear array probe was also used to detect the thyroid nodules, and the frequency was set to 5–8 MHz. Each patient was placed in the supine position while lying on the examination bed. Then, the neck was fully extended, and the patient was told to breathe calmly. The thyroid gland and its surrounding tissues and lymph nodes were examined. The number of nodules and the size, boundary, internal structure, internal echo, calcification and status of the lymph nodes was observed and recorded. Nodules that were solid, hypoechogenic, irregularly shaped, or taller than wide or contained microcalcification were considered suspicious for malignancy [20, 21]. According to the American Head and Neck Society and the American Academy of Otolaryngology-Head and Neck Surgery, the cervical lymph nodes were divided into six levels [22], and the abnormal lymph nodes were recorded by an ultrasound report.

### Radiomics analysis

The ultrasound images were exported from our imaging system, and the images were converted from jpg format to Digital Imaging and Communications in Medicine format. Then, we used ITK-SNAP software (http://www.itksnap.org/pmwiki/pmwiki.php) to draw an outline of the area of interest (ROI). The ROI was delineated by two ultrasound radiologists with more than 5 years of experience in thyroid nodules in the largest size of the thyroid nodule. Ultrosomics (GE healthcare, version 1.1) was used to extract the radiomics features and build and evaluate the models. In total, 1079 radiomics features were extracted. Before analysing the radiomics features, the patients were randomly divided into the training and validation groups. Then, the collinearity feature was used to process the radiomics features. Then, hypothesis testing, the least absolute shrinkage and selection operator method and a principal component analysis were used to reduce the dimensionality of the radiomics features. Then, a decision tree, naive Bayes, KNN, a logistics regression, SVM, bagging, random forest, extremely randomized trees, AdaBoost and a gradient boosting decision tree were used to build the models. Fifty models were built, and a receiver operating characteristic (ROC) curve was applied to evaluate the models. The closer to 1 the area under the curve (AUC), the better the model.

### Statistical analysis

The statistical analysis was performed using SPSS 23.0 (SPSS Inc., Chicago, IL, USA). The continuous quantitative data are shown as the mean ± standard deviation. An independent two-sample Student’s t-test was conducted to compare the data displaying a normal distribution. The categorical variables are shown as percentages, and a chi-square analysis or Fisher`s exact test was applied, as appropriate, to compare the results. A P-value < 0.05 indicated a significant difference.

## Results

### Correlation between ultrasound features and LNM

**Fig. 1:**
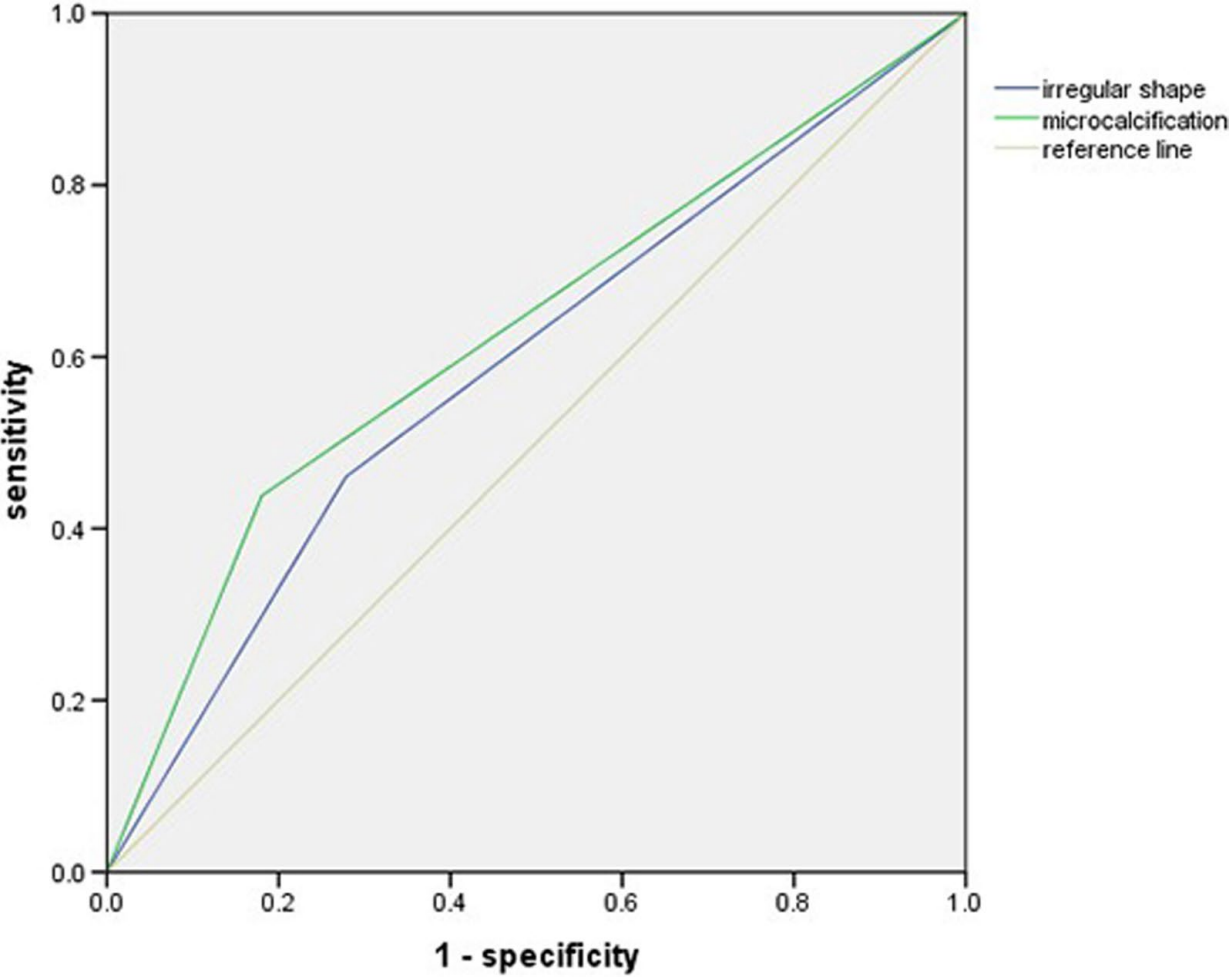
ROC curve analysis of the ultrasound characteristics of LNM in thyroid cancer patients. The AUC values of an irregular shape and microcalcification were 0.591 (P = 0.059) and 0.629 (P = 0.007), respectively

The relationship between the ultrasound features and LNM was analysed. Thyroid carcinoma with LNM tended to be irregularly shaped (P = 0.025) or contain microcalcifications (P = 0.004) (Table 1). However, other factors, such as solid-state, hypoechogenicity, and taller-than-wide features, did not significantly differ between the thyroid cancers with or without LNM. For the diagnosis of LNM, the ROC curve analysis suggested that the AUCs of the irregular shape and microcalcification were 0.591 (P = 0.059) and 0.629 (P = 0.007), respectively (Fig. 1).

**Table 1:** The relationships between LNM and ultrasound characteristics of thyroid cancer patients

		LNM		
		No	Yes	P
Composition	Spongiform	3 (37.5%)	5 (62.5%)	0.851
	Solid	58 (40.8%)	84 (59.2%)	
Echogenicity	Hyper- or equal-echogenicity	6 (27.3%)	16 (72.7%)	0.166
	Hypo-echogenicity or marked hypo-echogenicity	55 (43.0%)	73 (57.0%)	
Margin	Regular	44 (47.8%)	48 (52.2%)	0.025
	Irregular	17 (29.3%)	41 (70.7%)	
Shape	Non-ovoid	55 (42.3%)	75 (57.7%)	0.297
	Taller than wide	6 (30.0%)	14 (70.0%)	
Calcification	Macrocalcification or egg shell calcification or non calcification	50 (50.0%)	50 (50.0%)	0.004
	Microcalcification	11 (22.0%)	39 (78.0%)

### Evaluation of LNM of thyroid cancer by radiomics analysis

**Fig. 2:**
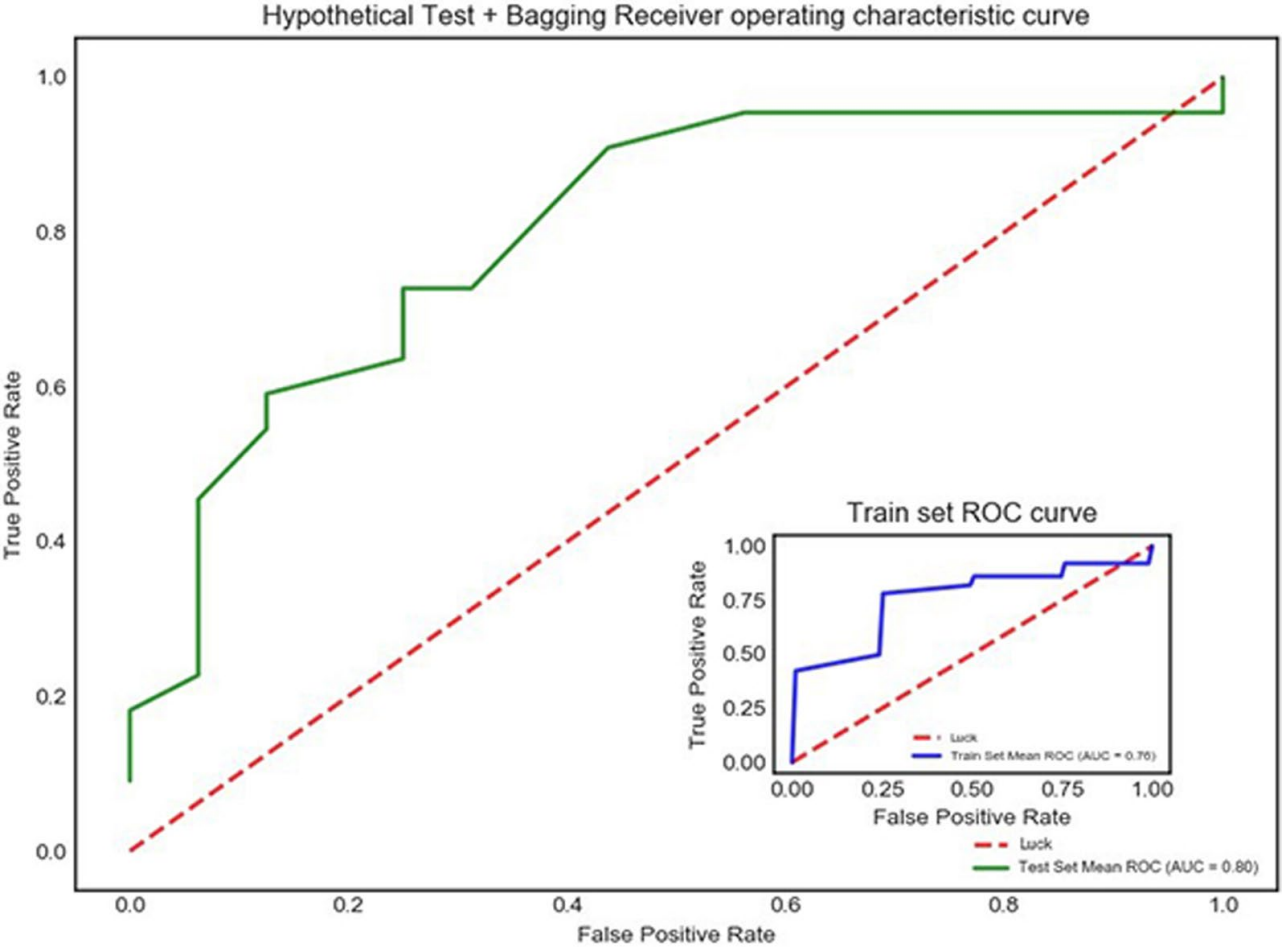
ROC curve analysis of the radiomics analysis of LNM in thyroid cancer patients. In the training set, the AUC value of LNM was 0.759. In the validation set, the AUC was 0.803

In total, 1079 parameters were obtained by Ultrosomics software, and only 690 parameters were included in the following step of the analysis after the non-colinear feature processing. A model built by hypothesis testing and bagging was selected to evaluate LNM. In addition, 91 radiomics features were included in this model. Significant differences were observed between LNM and the radiomics features. In the training set, the AUC value of LNM was 0.759, with a sensitivity of 0.90 and a specificity of 0.860. In the verification set, the AUC was 0.803, with a sensitivity of 0.727 and a specificity of 0.800 (Fig. 2).

## Discussion

Among all imaging methods, ultrasound is considered the most convenient method for assessing the characteristics of thyroid nodules and LNM. However, it is difficult to diagnose LNM involving small and nontypical lymph nodes in thyroid cancer in daily practice. Therefore, in this study, we focused on the clinical value of ultrasound features and radiomics analysis in the prediction of LNM in thyroid cancer. The current study showed that ultrasound features can predict LNM in thyroid cancer, but the meaningful features only included microcalcification and an irregular shape. In the present study, microcalcification and an irregular shape were associated with LNM, which is consistent with the results of previous studies [23, 24].

Although ultrasound is a recommended method for the detection of thyroid nodules and lymph nodes in clinical practice, it also has limitations. Radiomics analysis has great value in medicine in diagnosis and prognosis. Additionally, models built based on imaging can be used to predict LNM in many types of cancers. For example, a nomogram incorporating the radiomics signature and CA19-9 level can preoperatively predict LNM in patients with intrahepatic cholangiocarcinoma [25]. In other types of cancer, dynamic contrast-enhanced magnetic resonance imaging can also preoperatively predict sentinel LNM in breast cancer [26]. In addition, radiomics analysis based on 18 arterial-phase CT images has favourable predictive accuracy in LNM in patients with bladder cancer [27]. Therefore, the above studies highlight the feasibility of applying radiomics to assess malignant ultrasound features to predict LNM in thyroid cancer.

A meta-analysis demonstrated the value of ultrasound in the prediction of LNM in thyroid cancer with a sensitivity of 71% and specificity of 85% [28]. In this study, we used a radiomics analysis to predict LNM in patients with thyroid cancer on ultrasound. The findings of our study showed that the model had high sensitivity and specificity in predicting LNM, even higher than that of ultrasound [28], suggesting that radiomic analysis has great success in predicting LNM in thyroid cancer. Studies have demonstrated that radiomics analysis based on ultrasound is a promising way to assess the risk of LNM in thyroid cancer [16, 29]. In addition, a study showed that computer-aided diagnosis of the localization and differentiation of LNM from thyroid cancer on ultrasound had an accuracy of 83.0%, a sensitivity of 79.5% and specificity of 87.5% [30]. In addition to the application of ultrasound imaging radiomics, deep learning with CT can be used to predict LNM in thyroid cancer. A deep learning-based computer-aided diagnosis system had high accuracy in detecting LNM on preoperative CT in patients with thyroid cancer [31]. Based on this information, radiomics analysis has promising value in the prediction of LNM in thyroid cancer by ultrasound.

However, the present study has several limitations. This study was a retrospective study. In addition, the sample size of the population was small. The patients in this study were recruited from a single centre. In the future, large, multi-centre clinical studies are needed to further confirm the findings of this study.

## Conclusions

In conclusion, in thyroid carcinoma, microcalcification and an irregular shape are reliable predictors of LNM. Radiomics analysis has promising value in screening meaningful ultrasound features in thyroid cancer patients with LNM. Therefore, the prediction of LNM based on malignant ultrasound features and radiomic features is useful for making appropriate surgical decisions before surgery in clinical practice.

## Data Availability

The datasets analysed during the current study are available from the corresponding author on reasonable request. The confidential patient data should not be shared.

## Abbreviations

LNM: Lymph node metastasis
FNAB: Fine-needle aspiration biopsy
CT: Computed tomography
ROC: Receiver operating characteristic
AUC: Area under the curve
ROI: Area of interest
